# Evaluating the Protein Value of Fresh Tropical Forage Grasses and Forage Legumes Using In Vitro and Chemical Fractionation Methods

**DOI:** 10.3390/ani11102853

**Published:** 2021-09-29

**Authors:** Khaterine C. Salazar-Cubillas, Uta Dickhoefer

**Affiliations:** 1 Institute of Agricultural Sciences in the Tropics (Hans-Ruthenberg-Institute), Animal Nutrition and Rangeland Management in the Tropics and Subtropics, University of Hohenheim, 70599 Stuttgart, Germany; khaterine.salazar-cubillas@uni-hohenheim.de; 2 Institute of Animal Nutrition and Physiology, Christian-Albrechts-Universität zu Kiel, 24118 Kiel, Germany

**Keywords:** feed evaluation, post-ruminal protein, protein fractionation, tropical forages, ruminants

## Abstract

**Simple Summary:**

Various methods for estimating post-ruminal crude protein supply have been developed for temperate ruminant feedstuffs. However, their adequacy (i.e., accuracy and precision) to predict the post-ruminal crude protein supply of tropical forages is still questioned. Therefore, the objectives of the present study were: (1) to assess the adequacy of the in vitro and chemical methods to predict post-ruminal crude protein supply from fresh tropical forage, and (2) to identify nutritional composition variables that can predict post-ruminal crude protein supply. The in vitro method can estimate post-ruminal protein supply in tropical forages with moderate to high but not very slow passage rates. Available regression equations developed for temperate ruminant feedstuffs were not adequate enough to predict the post-ruminal protein supply of tropical forages. Instead, equations developed in the present study appear to predict the post-ruminal protein supply of tropical forages with reasonable adequacy.

**Abstract:**

The objectives of the present study were (1) to assess the adequacy of the in vitro and chemical methods to predict post-ruminal crude protein supply (PRCP) from fresh tropical forage, and (2) to identify PRCP supply predictors. Twenty-three fresh forage grasses and 15 forage legumes commonly used in domestic cattle feeding in the tropics and subtropics were incubated in the rumen of cows to determine ruminal crude protein (CP) degradation. The PRCP supply was calculated from in situ rumen-undegraded CP and in vitro organic matter digestibility (i.e., reference method), from ammonia-nitrogen release during in vitro incubation (i.e., in vitro method), and from the concentrations of chemical CP fractions (i.e., chemical method). The adequacy was evaluated using error-index and dimensionless parameters, and stepwise regression was used to select PRCP predictors. Adequacy ranged from poor to moderate (0.53 to 0.74) for the in vitro method being lower for forage legumes at a slow rumen passage rate (0.20), and even poorer (0.02 to 0.13) for the chemical method. Hence, the in vitro method can estimate PRCP supply in tropical forages with moderate to high but not with slow passage rates. Equations developed in the present study appear to predict PRCP supply with reasonable adequacy.

## 1. Introduction

Freshly cultivated forages are a major source of protein for domestic ruminants, particularly in the tropics and subtropics. The amount of rumen-undegraded feed crude protein (RUP) and microbial crude protein (CP) leaving the rumen are key variables in assessing their protein value. According to the German feeding recommendation system [1], the sum of RUP and microbial CP at the duodenum of ruminants is defined as post-ruminal crude protein (PRCP; formerly referred to as utilizable CP).

The PRCP supply to the small intestine has been studied for temperate ruminant feedstuffs using in vivo and in situ methods; however, these methods are costly, time-consuming, require ruminally and duodenally fistulated animals, and thus compromise animal welfare [2,3], rendering these methods unsuitable for routine evaluation of ruminant feedstuffs in tropical husbandry systems. Alternative methods such as the in vitro method developed by Steingaβ et al. [4] and the chemical method proposed by Zhao and Cao [5] have been tested in a wide range of temperate ruminant feeds; however, the adequacy (i.e., accuracy and precision; [3,6,7,8]) of these methods to predict the PRCP supply of common feedstuffs used in tropical husbandry systems is still questioned.

Forages grown in tropical regions differ in their chemical composition [9] and are characterized by a slower rate and lower extent of carbohydrate and CP degradation in the rumen than forages grown in temperate regions [10], which may hamper the estimation of PRCP supply with the in vitro and chemical methods. In the present study, it was therefore hypothesized that accuracy and precision of the in vitro method in estimating the PRCP supply from tropical forages might be poor due to early microbial lysis in the blank and higher rate of ammonium-nitrogen (NH_3_-N) uptake than release in the early stage of incubation of the feed samples [8], the latter being more pronounced in tropical than in temperate forages, because of their slow rate and low extent of carbohydrate and CP degradation in the rumen [10].

Moreover, it was hypothesized that the precision and accuracy of the PRCP supply predicted from the CP fractions using the only available equation of Zhao and Cao [5] for dried forage grasses, a grass silage, a fresh forage legume, and corn and soybean by-products are most likely poor and lower than that of the in vitro method, because forage samples (i.e., forage grasses and forage legumes) were not representative of common forages used for domestic cattle feeding in the tropics and subtropics, and the relationships between chemical CP fractions and PRCP supply might be different between tropical and temperate forages. 

Therefore, the objectives of the present study were (1) to assess the adequacy of the PRCP supply of fresh tropical forage grasses and forage legumes estimated with the in vitro and chemical methods, and (2) to identify nutritional composition variables and develop specific algorithms that can be used to predict the PRCP supply of fresh tropical forages commonly used in domestic cattle feeding in the Tropics and Subtropics.

## 2. Materials and Methods

Detailed information on the collection and origin is described in Appendix A. All animal handling and procedures were performed following the Animal Welfare Legislation approved by the Government Presidium of Stuttgart, Germany (approval code V319/14 TE).

### 2.1. Proximate Nutrient and Fiber Analysis

The proximate nutrient and chemical fiber fractions of the forage samples were analyzed in duplicate according to the German Handbook of Agricultural Experimental and Analytical Techniques [11] and then mean values of duplicate measurements were reported in Table 1. The dry matter (DM) concentration of the forage samples was determined by drying the forage samples in a forced-air oven (F115, Binder GmbH, Tuttlingen, Germany) at 103 °C until constant weight (Method 3.1). The remaining feed substrate after drying was weighed and incinerated in a muffle furnace (D-2804, Nabertherm GmbH, Bremen, Germany) at 550 °C for 5 h to determine the crude ash (CA) concentration (Method 8.1).

The nitrogen (N) concentration of the forage samples was determined by the Kjeldahl method using a distillation apparatus (B324, Büchi Labortechnik GmbH, Essen, Germany) and then converted to CP by multiplying it by 6.25 (method 4.1.1).

The neutral-detergent fiber concentration assayed using heat-stable amylase and sodium sulfite and expressed inclusive of residual CA (aNDF) was determined in an ANKOM Fiber Analyzer (A200, ANKOM Technology, NY, USA; Method 6.5.1). The remaining substrate after aNDF analysis was treated with an acid-detergent solution in an ANKOM Fiber Analyzer to determine the acid-detergent fiber concentration expressed inclusive of residual CA (ADF; Method 6.5.2). Thereafter, the remaining substrate was rinsed with a sulfuric acid solution in a 500 mL beaker to determine the acid-detergent lignin (Lignin_(sa)_) concentration (Method 6.4.1).

### 2.2. Reference Post-Ruminal Protein Estimation

The reference PRCP supply was estimated using the equation N°11 of Lebzien et al. [15] at rumen passage rate (Kp) of 2, 5, and 8%/h taking into consideration that in tropical areas, animals with very low to low feed intake level and low-yielding (i.e., slow Kp), as well as high-yielding dairy cows can be found (i.e., fast Kp).

PRCP = [187.7 − (115.4 × (RUP/CP))] × DOM + 1.03 × RUP;(1)

where PRCP is the PRCP supply (g/kg DM) at Kp of 2, 5, and 8%/h; RUP is the RUP concentration (g/kg DM) estimated with the in situ method at Kp of 2, 5, and 8%/h; CP is the CP concentration of the original forage sample (g/kg DM); DOM is the digested organic matter concentration (g/1000 g DM).

The rumen in situ CP degradation kinetics were determined following the Madsen and Hvelplund [16] protocol with incubation times of 2, 4, 8, 16, 24, 48, and 72 h during two periods with three cows per period. The CP disappearance at each incubation time was corrected for losses of water-soluble feed CP and water-insoluble feed CP escaping the bag in the form of small particles using the equation suggested by Weisbjerg et al. [17]. The CP disappearance at each incubation time was corrected for microbial attachment to undegraded feed particles using the equation of Krawielitzki et al. [18]. Then, CP degradability at Kp of 2, 5, and 8%/h was estimated using the equation of Dhanoa et al. [19] and RUP was estimated as the concentration of CP minus the concentration of rumen-degraded CP.

The DOM (g/1000 g DM) was estimated by multiplying digested organic matter (dOM; g/1000 g organic matter) by the organic matter concentration (g/kg DM) of the forage sample and divided by 1000. The dOM was estimated using the equation N°43e of Menke and Steingass [14].

dOM = (15.38 + 0.85 × GP + 0.06 × CP + 0.07 × CA) × 10;(2)

where dOM is the dOM proportion (g/1000 g organic matter); GP is the net gas release after 24 h in vitro incubation (mL/200 mg DM of the original feed substrate); CP is the CP concentration of the original forage sample (g/kg DM); CA is the CA concentration of the original forage sample (g/kg DM). The GP was estimated following procedures of the regular Hohenheim gas test [14].

GP_24_ = (V_24_ − V_0_ − GP_0_) × 200 × CF/W; (3)

where GP_24_ is the net gas release after 24 h in vitro incubation of the original feed substrate (mL/200 mg DM of the original feed substrate); V_24_ is the position of the piston after 24 h in vitro incubation of the syringe containing feed substrate and inoculum (mL); V_0_ is the position of the piston at the beginning of the incubation of the syringe containing feed substrate and inoculum (mL); GP_0_ is the mean gas release after 24 h in vitro incubation of the three syringes containing only inoculum (mL; i.e., blanks); CF is the mean correction factor of the three syringes containing hay standard and the three syringes containing concentrate standard sample material (from 0 to 1; i.e., standard of the University of Hohenheim); W is the weight of the original feed substrate of the syringe containing feed substrate and inoculum (mg DM).

The GP_24_ of the hay and concentrate standards were used to correct the net gas release of each forage sample in the same incubation run. For this, the reference GP_24_ of the hay and concentrate standard was divided by the mean GP_24_ of the three syringes containing hay and concentrate standard sample material, respectively. Runs were repeated if these correction factors were <0.9 or >1.1. 

Three GP_24_ for each forage sample were calculated for each run. A maximum 10% coefficient of variation (CV; expressed as a percentage of the overall mean) was allowed in GP_24_ between and within runs. The mean of at least five repetitions of GP_24_ represented the GP_24_ of each forage sample.

### 2.3. Modified Hohenheim Gas Test

The PRCP supply of all feedstuffs was estimated in two or three runs with three repetitions per incubation time in each run. Incubation times were 8 and 48 h following the recommendations of Leberl et al. [20]. 

Rumen fluid was collected with a vacuum pump from two or three fistulated cows, including those used for the in situ incubation. The rumen fluid was extracted before morning feeding and transported to the laboratory in prewarmed thermal flasks, where it was first filtered through a cloth layer with a pore size of 100 µm. Of the filtered rumen fluid, 420.6 mL was taken and added to 841.1 mL of a prewarmed colorless incubation solution (~39 °C) to generate the inoculum for the in vitro incubations. The incubation solution was prepared following the procedure of the regular Hohenheim gas test [14] with a chemical alteration of 2 g/L increase in ammonium bicarbonate and 2 g/L decrease in sodium bicarbonate. The incubation solution (841.1 mL) of the modified Hohenheim gas test was prepared in the following order: 400 mL distilled water, 0.1 mL micromineral solution (13.2 g calcium chloride × 2 H_2_O, 10 g manganese chloride × 4 H_2_O, 1 g cobalt chloride × 6 H_2_O, 8 g ferric trichloride × 6 H_2_O, and made up to 100 mL with distilled water), 200 mL buffer solution (6 g ammonium bicarbonate, 33 g sodium bicarbonate, and made up to 1000 mL with distilled water), 200 mL macro-mineral solution (5.7 g disodium hydrogen phosphate, 6.2 g potassium dihydrogen phosphate, 0.6 g magnesium sulfate × 7 H_2_O, and made up to 1000 mL with distilled water), 1 mL resazurin solution (0.1%, 100 mg resazurin in 100 mL of distilled water), and 40 mL freshly prepared reduction solution (4 mL sodium hydroxide 1N, 625 mg sodium sulfide × 9 H_2_O, and 95 mL distilled water). The incubation solution and later the inoculum were stirred with a magnetic stir and kept under a continuous flux of carbon dioxide in a water bath at ~39 °C.

After 5 min of homogenization, 30 mL of the inoculum was added to each prewarmed syringe (~39 °C) containing approximately 200 mg DM of forage sample material. Per incubation time, three syringes containing only inoculum (i.e., blanks) and three syringes containing a standard protein sample material (i.e., protein standard of the University of Hohenheim) were additionally included in each run. Syringes were randomly placed in a prewarmed water bath (~39 °C) and were shaken every hour during the first 6 h of incubation. 

Immediately after 8 and 48 h of incubation, all contents of the respective syringes were transferred to a 50 mL sterile plastic tube and stored (4 °C) until the next day for analysis. Then, two subsamples of 10 mL each of the content of each syringe were transferred into two Kjeldahl flasks and 10 mL of 0.25 M phosphate buffer with a pH of 11 was added to each flask to increase the pH of the sample solution. Immediately thereafter, the NH_3_-N release from the inoculum of the blanks and syringes containing forage samples or protein standard was then estimated with back titration using a 0.05 M sulfuric acid solution.

The mean NH_3_-N release from the two 10 mL aliquots for each syringe containing the blank, the forage sample, or the protein standard was multiplied by three to calculate the NH_3_-N release from 30 mL of inoculum. Each triplicate measurement of NH_3_-N release in 30 mL of the syringes containing the blank, the forage sample, or the protein standard after 8 and 48 h in vitro incubation was then used to calculate the PRCP supply after 8 and 48 h in vitro incubation using the equation of Steingaβ et al. [4]:

PRCP = ((N sample + NH_3_-N blank − NH_3_-N sample)/W) × 1000 × 6.25; (4)

where PRCP is the PRCP supply of the forage samples or protein standard after 8 or 48 h in vitro incubation (g/kg DM); N sample is the N concentration of the original forage sample or protein standard incubated in 30 mL of inoculum (mg/30 mL inoculum); NH_3_-N blank is the NH_3_-N release from the blank after 8 and 48 h in vitro incubation (mg/30 mL inoculum); NH_3_-N sample is the NH_3_-N release from the forage sample or protein standard after 8 or 48 h in vitro incubation (mg/30 mL inoculum); W is the initial weight of the original forage sample or protein standard incubated in 30 mL inoculum (mg DM/30 mL inoculum). 

The PRCP supplies of the protein standard were used to correct the PRCP supply after 8 and 48 h in vitro incubation. For this, the reference PRCP supply of the protein standard at each in vitro incubation time was divided by the mean PRCP supply of the three syringes containing the protein standard after 8 and 48 h. Runs were repeated if these correction factors were <0.9 or >1.1. Then, each PRCP supply after 8 and 48 h in vitro incubation of the forage samples was multiplied by the respective correction factor. 

The PRCP supply at Kp of 2, 5, and 8%/h was obtained by plotting the log of the time of incubation (i.e., ln(8) and ln(48)) against PRCP supply after 8 and 48 h in vitro incubation, respectively. From the resulting non-linear regression equation, the intercept and slope were obtained. The PRCP supply was then calculated using the equation presented by Edmunds et al. [3]:

PRCP = a × ln (1/Kp) + b;(5)

where PRCP is the PRCP supply at Kp of 2, 5, and 8%/h of the forage sample (g/kg DM); a is the slope (g/kg DM); Kp is the assumed Kp expressed as 2, 5, and 8%/h; b is the intercept (g/kg DM).

Three PRCP supplies for each Kp were calculated for each run. A maximum 10% CV (expressed as a percentage of the overall mean) was allowed in PRCP supplies at a given Kp between and within runs. The mean of at least five repetitions of PRCP supplies was calculated for each Kp, representing the PRCP supply at a given Kp of each forage sample.

### 2.4. Chemical Crude Protein Fractionation

The non-protein N (NPN) concentration was determined in duplicate using the tungstic acid method [21]. The forage sample material was weighed into a 100 mL flask, and then 50 mL of cold distilled water and 8 mL of a 0.3 M sodium tungstate solution were added. The forage sample material and solution were mixed for 30 min under continuous stirring before reducing the pH to 2.0 with a 0.5 M sulfuric acid solution. Flasks were then covered and kept at room temperature for 16 h. Then, the suspension was filtered through cellulose filter paper (Whatman paper N°54, GE Healthcare Life Sciences, Darmstadt, Germany). Both the filter paper and residual substrate were washed once with 250 mL of cold distilled water before they were analyzed for N. Then, the NPN concentration was calculated by subtracting the N amount in the residual substrate and the N amount in the cellulose filter paper from the total N amount in the original forage sample material. 

The soluble true protein (SP) concentration was determined in duplicate following Licitra et al. [13] recommendations. Briefly, 50 mL of a borate-phosphate buffer (pH 6.7–6.8) [22] and 1 mL of freshly prepared sodium azide 1.5 M were added to a 100 mL flask containing forage sample material. Flasks were covered for 3 h before the mixture was filtered through cellulose filter paper. Both the filter paper and residual substrate were washed once with 250 mL of cold distilled water before both were analyzed for N. The SP concentration was calculated by subtracting the N amount in the residual substrate and the N amount in the cellulose filter paper from the total N amount in the original forage sample material.

The concentration of neutral-detergent-insoluble protein (NDIP) was determined in duplicate following the procedures of aNDF analysis without the use of sodium sulfite [13]. The forage sample material was boiled in a 500 mL beaker with 100 mL of neutral-detergent solution [23] using a laboratory heater (EV1, Gerhardt GmbH & Coerhardt, Königswinter, Germany). After the solution started boiling, 25 μL of alpha-amylase was added each at 1 min and 30 min. One hour after the solution started boiling, the mixture was filtered through cellulose filter paper. Both the filter paper and residual substrate were washed once with 250 mL of hot distilled water (~80 °C), rinsed twice with 5 mL of acetone, and dried at room temperature for 1 h before they were analyzed for N. The analysis of acid-detergent-insoluble protein (ADIP) followed the same procedure as NDIP, except that the neutral-detergent solution was substituted for an acid-detergent solution and alpha-amylase was not used.

In addition to CP fraction analyses, concentrations of aNDF and ADF estimated from the residue after boiling in the respective solution without the use of sodium sulfite according to Licitra et al. [13] were also determined, herein referred to as NDFp and ADFp, respectively.

The N concentrations of the residual substrate and cellulose filter after the chemical CP fractionation procedure were determined using method 4.1.1 [11] as described in Section 2.1. The means of the duplicate measurements of the different chemical CP fractions of the forage samples were then used to calculate the CP fractions as described by Sniffen et al. [12]:

(6) $$\begin{array}{c} A=\mathrm{NPN}\times 6.25;\\ B_{1}=\mathrm{SP}-(\mathrm{NPN}\times 6.25);\\ B_{2}=\mathrm{IP}-\mathrm{NDIP};\\ B_{3}=\mathrm{NDIP}-\mathrm{ADIP};\\ C=\mathrm{ADIP}. \end{array}$$

where A is the concentration of CP soluble in the borate-phosphate buffer and tungstic acid solution (g/kg DM); NPN is the concentration of NPN-N (g/kg DM); B_1_ is the concentration of true protein soluble in buffer solution and precipitated by the tungstic solution (g/kg DM); SP is the concentration of SP (i.e., sum of CP fractions A and B_1_; g/kg DM); B_2_ is the concentration of true protein insoluble in buffer solution but soluble in the neutral-detergent solution (g/kg DM); IP is the concentration of insoluble true protein estimated as the concentration of CP minus the concentration of SP (i.e., sum of true protein fractions B_2_, B_3_, and C; g/kg DM); NDIP is the concentration of NDIP known as cell-wall-bound true protein (i.e., sum of true protein fractions B_3_ and C; g/kg DM); B_3_ is the concentration of true protein soluble in acid-detergent solution but insoluble in neutral-detergent solution (g/kg DM); ADIP is the concentration of ADIP (g/kg DM); C is the concentration of true protein insoluble in the acid-detergent solution (g/kg DM). 

The CP not bound to the cell wall (i.e., sum of CP fractions A, B_1_, and B_2_) and the true protein (i.e., sum of true protein fractions B_1_, B_2_, B_3_, and C) were also calculated.

The PRCP supply was estimated from the concentrations of chemical CP fractions using the only available equation for dried forage grasses, a grass silage, a fresh forage legume, and corn and soybean by-products [5]:

PRCP = 8.78 × A + 15.69 × B_1_ + 12.36 × B_2_ + 11.83 × B_3_ + 6.99 × C (7)

where PRCP is PRCP supply after 24 h in vitro incubation (g/kg DM); A is the concentration of CP soluble in the borate-phosphate buffer and tungstic acid solution (g/kg DM); B_1_ is the concentration of true protein soluble in buffer solution and precipitated by the tungstic solution (g/kg DM); B_2_ is the concentration of true protein insoluble in buffer solution but soluble in the neutral-detergent solution (g/kg DM); B_3_ is the concentration of true protein soluble in acid-detergent solution but insoluble in neutral-detergent solution (g/kg DM); C is the concentration of true protein insoluble in the acid-detergent solution (g/kg DM).

### 2.5. Statistical Analyses

All statistical analyses were conducted using R statistical software version 3.6.1 (R Core Team, Vienna, Austria). The means of the duplicate measurements per sample of proximate nutrients, chemical fiber fractions, CP fractions, fermentation parameters after 24 h in vitro incubation, and PRCP supply as estimated with Lebzien et al. [15] equation (i.e., reference method) of fresh tropical forage grasses (n = 23) and forage legumes (n = 15) were calculated and described using descriptive statistics including measures of central tendency (i.e., mean and median) and measures of variability and dispersion (i.e., minimum, maximum, and standard deviation). 

Previous to the adequacy assessment, a Grubbs outlier test [24] was performed to identify illogical values in the sample set of PRCP supply as estimated with the in vitro, chemical method, and reference method. The outlier test identified one outlier in the PRCP supply estimated with the in vitro method at Kp of 2%/h: Centrosema sp (DC.) Benth (179 g/kg CP). However, the outlier was not removed from the sample set, because the identified outlier was not a common-sense outlier (i.e., illogical value).

To evaluate the adequacy of the predictions of the in vitro and chemical methods, the estimates of PRCP supply at Kp of 2, 5, and 8%/h from the in vitro method and chemical method at Kp of 5%/h were evaluated against values determined by the reference method using error-index and dimensionless parameters. The estimates of PRCP supply according to the chemical method were evaluated only at Kp of 5%/h because the equation of Zhao and Cao [5] was developed to predict the PRCP supply after 24 h in vitro incubation, which resembles a PRCP supply at Kp of 5%/h.

The error-index parameters included the mean bias, root mean square error (RMSE), and mean absolute percentage error (MAPE), whereas dimensionless parameters included the RMSE to standard deviation ratio (i.e., RSR), and the concordance correlation coefficient (CCC). The CCC as a combined measure of accuracy and precision was calculated and partitioned into the correlation coefficient (i.e., precision; ρ) and a bias correction factor coefficient (i.e., accuracy; Cb) [25].

The scale of McBride [26] was used to assess the degree of agreement between the alternative method and the reference method, which classifies the CCC as very strong (CCC ≥ 0.90), strong (CCC ≥ 0.80–<0.90), moderate (CCC ≥ 0.65–< 0.80), and poor (CCC < 0.65). A more accurate and precise prediction was considered to be the one with lower mean bias, RMSE, MAPE, RSR, and greater CCC. In the present study, an alternative method was considered adequate enough to replace the reference method, if the CCC was ≥ 0.80 because CCC estimates between PRCP estimated with the in vitro and in vivo methods had ranged from 0.81 to 0.89 in a previous study [6]. In addition, the scale of Evans [27] was used to classify the correlation as very strong (*p* ≥ 0.80), strong (*p* ≥ 0.60–< 0.80), moderate (*p* ≥ 0.40–< 0.60), weak (*p* > 0.20–< 0.40), and very weak (*p* ≤ 0.20; objective 1). 

According to previous studies on CP degradation in the rumen, concentrations (g/kg DM) of proximate nutrients (i.e., CA and CP), chemical fiber fractions (i.e., aNDF, ADF, NDFp, ADFp, and Lignin_(sa)_), and CP fractions (i.e., A, B_1_, B_2_, B_3_, C, SP, IP, true protein, NPN, cell-wall-bound protein and CP not bound to the cell wall), as well as the ratios between concentrations of chemical CP fractions (i.e., SP/IP, IP/SP, true protein/NPN, NPN/true protein, cell-wall-bound true protein/CP not bound to the cell wall and CP not bound to the cell wall/cell-wall-bound true protein), were selected as a set of independent variables that can predict the PRCP supply of tropical forage grasses and forage legumes. 

An attempt was made to develop one equation per Kp (i.e., 2, 5, and 8%/h) and per forage type (i.e., forage grasses and forage legumes), but the PRCP supply was better predicted with a general equation across both forage types rather than for forage grasses and forage legumes separately. Therefore, three equations (i.e., one equation per Kp) were developed with independent and dependent variables expressed in g/kg DM using a multiple linear regression forward and backward stepwise approach with Akaike Information Criteria as model selection criteria. In the case that several models were obtained per Kp with the stepwise multiple linear regression approach, the model with the lowest Bayesian Information Criterion was selected. Finally, multicollinearity and independence of residuals of the selected model were evaluated using variance inflation factor and residual plots, respectively. Independent variables with variance inflation factor > 10 were removed from the model until the variance inflation factor of the remaining independent variables was <10 [28]. 

The standard error of the mean, *p*-value, determination coefficients adjusted by the number of predictors in the model (adjusted R^2^), RMSE, and MAPE were calculated from the relationship between PRCP supply estimated with the reference method and those predicted with the developed equations in the present study for tropical forages at Kp of 2, 5, and 8%/h (objective 2).

## 3. Results

### 3.1. Nutritional Characteristics of Forages

Descriptive statistics of the chemical composition, CP fractions (i.e., A, B_1_, B_2_, B_3_, and C), in vitro fermentation parameters, and PRCP supply as estimated with the reference method of fresh tropical forage grasses and forage legumes are presented in Table 1.

For fresh forage grasses, the concentrations of SP, IP, and true protein ranged from 19 to 103 g/kg DM, from 27 to 104 g/kg DM, and from 30 to 107 g/kg DM, respectively. The concentrations of cell-wall-bound true protein and CP not bound to the cell wall ranged from 12 to 64 g/kg DM and from 35 to 140 g/kg DM, respectively. 

For fresh forage legumes, the concentrations of SP, IP, and true protein ranged from 36 to 93 g/kg DM, from 90 to 164 g/kg DM, and from 92 to 168 g/kg DM, respectively. The concentrations of cell-wall-bound true protein and CP not bound to the cell wall ranged from 16 to 98 g/kg DM and from 60 to 188 g/kg DM, respectively. 

### 3.2. Adequacy of the In Vitro Method to Predict PRCP Supply

For all comparisons, greater CCC estimates complied with lower RMSE, MAPE, and RSR. 

The PRCP supply of tropical forages was poorly predicted by the in vitro method at Kp of 2%/h (CCC = 0.53), but moderately predicted at Kp of 5%/h (CCC = 0.69) and 8%/h (CCC = 0.74; Table 2). The precision (ρ from 0.53 to 0.74) to predict reference PRCP supply of tropical forages by the in vitro method increased as Kp increased (Kp from 2 to 8%/h), whereas the accuracy was similar across Kp (Cb from 0.82 to 0.84).

The range of PRCP supply determined with the in vitro method was wider (81 to 171 g/kg DM) than that of values estimated with the equation from Lebzien et al. [15] (39 to 185 g/kg DM) for our sample set.

The PRCP supply of forage grasses was poorly predicted at Kp of 2%/h but moderately predicted at Kp of 5 and 8%/h, whereas the PRCP supply of tropical forage legumes was poorly predicted by the in vitro method for all Kp. 

The PRCP supply estimated using the in vitro method slightly underestimated (mean bias from 5.90 to 8.61 g/kg DM) the PRCP supply determined with the reference method of tropical forage grasses for all Kp and of forage legumes at Kp of 2%/h (mean bias of 4.05 g/kg DM), whereas it slightly overestimated (mean bias of from −9.87 to −6.03 g/kg DM) the PRCP supply of tropical forage legumes at Kp of 5 and 8 %/h (Table 2).

### 3.3. Adequacy of the Chemical Method to Predict PRCP Supply and Its Comparison with the In Vitro Method

**Figure 1:**
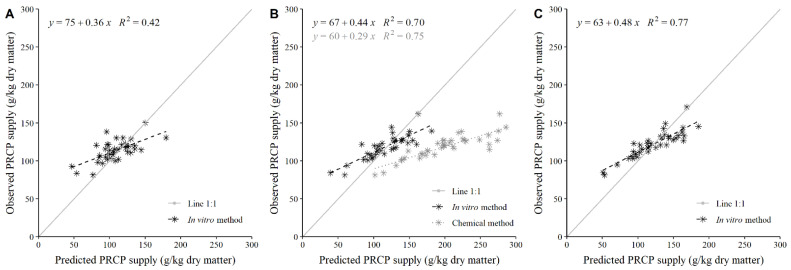
Relationship between post-ruminal protein (PRCP) supply of 23 fresh forage grasses and 15 fresh forage legumes that are commonly used in domestic cattle feeding in the tropics and subtropics estimated with a reference method [15] (observed PRCP) and with an in vitro method (i.e., modified Hohenheim gas test; predicted PRCP) evaluated at Kp of 2%/h (**A**), 5%/h (**B**), and 8%/h (**C**), or with a chemical method [5] at Kp of 5%/h (**B**).

Greater CCC estimates resulted in lower mean bias, RMSE, MAPE, and RSR. Irrespective of the forage type, the PRCP supply at Kp of 5%/h determined by the reference method was poorly predicted with the chemical method (CCC ≤ 0.14) using the equation of Zhao and Cao [5] (Table 2). The equation of Zhao and Cao [5] greatly overestimated (i.e., negative mean bias from −138.79 to −14.63 g/kg DM) the PRCP supply according to the reference method (Table 2; Figure 1B). Irrespective of the forage type, the poor adequacy of the equation of Zhao and Cao [5] was more related to its low accuracy (Cb from 0.05 to 0.16) and not a poor precision (ρ from 0.56 to 0.87; Table 2). 

Irrespective of the forage type, the PRCP supply at Kp of 5%/h determined by the reference method was better predicted by the in vitro method than by the chemical method with lower mean bias, RMSE, MAPE, and RSR, as well as greater CCC (Table 2).

Adequacy of the estimates of PRCP supply was overall greater (lower RMSE, MAPE, and RSR, and greater CCC) for forage grasses than for forage legumes for both the in vitro and the chemical method (Table 2).

### 3.4. Multivariate Regressions to Predict PRCP Supply in Tropical Forages

One equation per Kp was developed to predict the PRCP supply of both, tropical forage grasses and forage legumes (Table 3). The variance inflation factor was lower than 2.4 for all independent variables and the residual plots of the developed equations showed no clear patterns and revealed a similar distribution of plotted points around the line at 0 (Appendix B). 

The variables retained to predict the PRCP supply of tropical forages were IP and ADF, irrespective of the Kp. The adjusted R^2^, RMSE, and MAPE calculated from the relationship between PRCP supply according to the reference method and that predicted with the equations developed in the present study for tropical forages ranged from 0.80 to 0.85, 6.0 to 6.6 g/kg DM, and 4.3 to 4.4% of the reference PRCP supply, respectively.

## 4. Discussion

The PRCP supply of 23 forage grasses and 15 forage legumes that are commonly used in domestic cattle feeding in the tropics and subtropics was estimated at Kp of 2, 5, and 8%/h using the modified Hohenheim gas test as in vitro method and predicted from chemical CP fractions using the equation of Zhao and Cao [5] at Kp of 5%/h.

The present study aimed (i) at assessing the adequacy of these two approaches when compared to a reference method, for which RUP concentrations were determined in situ, and (ii) at identifying nutritional composition variables and develop specific algorithms for tropical forages to improve prediction of PRCP supply by the chemical method. 

### 4.1. Experimental Design and Methods

Besides a low reproducibility of the concentrations of different CP fractions during the lab analysis, one limitation of the present study may be related to the choice of reference method and its robustness. Since cows equipped with both, ruminal and duodenal fistula, were not available, the PRCP supply was derived from the RUP concentration of the forages as determined in situ, while the microbial CP was estimated from the DOM using an efficiency of microbial CP synthesis adjusted for the availability of rumen-degraded CP [15]. 

The PRCP estimated with the equation of Lebzien et al. [15] was chosen as a reference because, to our knowledge, a specific equation for tropical forages to calculate PRCP supply from concentrations of RUP and DOM or metabolizable energy concentrations has not been published. 

The great number of observations used to develop the equation of Lebzien et al. [15], the fact that the reference values were determined in in vivo studies using double-fistulated animals, and the strong relationship between dependent and independent variables (as indicated by high R^2^ and low CV) indicate that the equation of Lebzien et al. [15] might be able to predict with an acceptable margin of error the PRCP supply of diets and individual feedstuffs. 

Although this equation has been developed for temperate diets and individual feedstuffs, their range of diets included those of only forages (e.g., forage to concentrate ratios from 100:0 to 30:70) and with low CP concentrations (e.g., grass hay). In addition, the CP and RUP concentrations of our forage sample set were within the range of those of the diets used to develop the equation of Lebzien et al. [15]. Moreover, the efficiency of rumen microbial CP synthesis calculated for the forage samples in the present study using the equation of Lebzien et al. [15] ranged from 119 to 179 g microbial CP/kg DOM, which is similar to the efficiency values estimated for cattle in tropical environments (111 to 201 g microbial CP/kg DOM; n = 444 individual observations) [29] using the equations proposed by INRA [30]. Therefore, we expect that the equation of Lebzien et al. [15] can also adequately predict the PRCP supply of tropical forages. 

The DOM was estimated from the GP during in vitro incubation of forage samples, according to Menke and Steingass [14]. Similarly, the equation used to predict dOM [14] was developed using temperate feedstuffs, which might have affected estimates of the reference PRCP supply. However, the equation was developed based on in vivo digestibility data for a great variety of fresh and dry forages (n = 185). Furthermore, the CP concentration, in vitro GP, and thus dOM of our forage samples were within the range of those feedstuffs used to develop the equation of Menke and Steingass [14], suggesting that it can also adequately predict the dOM of tropical forages. 

The reference and in vitro PRCP methods were estimated at Kp of 2, 5, and 8%/h. Those Kp were chosen in the present study because they were considered appropriate to represent the range of Kp that can be found in the tropics and subtropics. This Kp range was also found in the dataset of Salazar and Dickhoefer [29] that summarizes 444 individual observations of steers, heifers, and lactating cows under tropical conditions and includes animals with very low feed intake levels (e.g., during dry seasons; Kp < 5%/h), low-yielding animals, as well as high-yielding dairy cows (i.e., >30 kg milk/day; Kp > 5%/h).

### 4.2. Nutritional Characteristics of Forages

The concentrations of proximate nutrients, fiber fractions, CP fractions, and the in vitro fermentation parameters (i.e., GP, DOM, and metabolizable energy) of most analyzed forage species were within the range of values described for the respective species in the literature [30,31,32,33,34,35,36,37,38]. No published information was available on the PRCP supply from tropical forages; however, the RUP concentrations determined in situ were within the range of values found in previous studies for the respective forage species [30,39,40,41,42,43,44,45,46,47,48]. Hence, in general, the forage samples included in the present study seem to be representative of tropical forage grasses and legumes used in domestic cattle feeding in tropical and subtropical countries.

### 4.3. Adequacy of the Estimates of Post-Ruminal Crude Protein Supply Using the In Vitro Method

It was expected that the in vitro method poorly predicts the PRCP supply from tropical forages as a result of its low accuracy and precision caused by the overestimation of NH_3_-N release of the blank and the underestimation of NH_3_-N release from the feed sample. The NH_3_-N release from the inoculum is overestimated because microbial lysis is greater in the blank than those syringes containing feed substrate due to a lack of fermentable substrates [8]. It is also possible that NH_3_-N release of the feed sample in an in vitro system is underestimated because NH_3_-N release and NH_3_-N uptake by microorganisms occur simultaneously [8] with a higher rate of uptake than release in the early stage of incubation [49]. 

Our hypothesis was partly accepted. The overall mean bias was low (mean bias from 1.17 to 5.17 g/kg DM), indicating great compliance between the PRCP supply of tropical forages as estimated according to Lebzien et al. [15] and with the in vitro method. Nevertheless, there were considerable and similar positive (from 0.16 to 53.60 g/kg DM) and negative biases (from −37.48 to −0.07g/kg DM) for individual forage samples, explaining the low mean bias. In this line relatively high RMSE (from 18.36 to 18.72 g/kg DM) and MAPE (from 15.12 to 15.99 g/kg DM) represent better the expected error of the in vitro method than the mean bias. Moreover, the in vitro method showed a poor to moderate agreement (CCC from 0.53 to 0.74), with lower CCC estimates at slow than at fast Kp and in tropical forage legumes than forage grasses. This poor to moderate level of agreement was related to a low precision (*p* = 0.65) rather than a low accuracy (Cb = 0.82). Hence, the precision of the in vitro method (*p* from 0.53 to 0.74) increased as Kp increased, whereas the accuracy was similar irrespective of the Kp (Cb from 0.82 to 0.84). 

Similarly, a poor to moderate level of agreement was found in Edmunds et al. [3] (n = 23 samples of fresh temperate and conserved forage grasses and legumes; CCC from 0.23 to 0.68) and Westreicher-Kristen et al. [7] (n = 13 samples of dried distillers’ grains with solubles; CCC from 0.35 to 0.44), between the PRCP supply estimated with the reference method [15] and the one derived with the in vitro method. In contrast thereto, predicted PRCP supply strongly agreed with the reference values in a study by Zhao and Lebzien [6] (n = 25 samples of conserved forages grasses, concentrates components, and concentrate mixtures; CCC from 0.81 to 0.89). 

The wider PRCP supply of the sample set of Zhao and Lebzien [6] (from 76 to 341 g/kg DM) and the in vivo reference PRCP method used (i.e., measured CP at the duodenum) could have contributed to reducing the uncertainty of the slope estimate (i.e., precision) [50] and might explain the greater adequacy of the in vitro method in their study. Additionally, Zhao and Lebzien [6] also used the equation of Lebzien et al. [15] as a reference method; however, their RUP concentration was estimated based on measured in vivo CP at the duodenum, measured microbial CP at the duodenum, and a fixed endogenous CP factor. Therefore, their reference PRCP supply was estimated indirectly from measured PRCP supply. 

The lower accuracy of predicting PRCP supply of forage legumes at fast Kp (i.e., short incubation time) than slow Kp (i.e., long incubation time) by the in vitro method can be explained by the prolonged lag phase presented in forage legumes (67 min) than in forage grasses (50 min) [51].

The greater adequacy of the in vitro method in tropical forage grasses than forage legumes might be related to the fact that protein and carbohydrate degradation is more synchronous, both, in amount and time, in tropical forage grasses than forage legumes (i.e., high CP and low potentially digestible aNDF) [52], allowing for an in vitro fermentation without at least temporal nutrient restrictions for microbial fermentation. 

The PRCP supply estimated using the in vitro method slightly underestimated (mean bias from 5.90 to 8.61 g/kg DM) the PRCP supply determined with the reference method of tropical forage grasses for all Kp and of forage legumes at Kp of 2%/h (mean bias of 4.05 g/kg DM). In contrast, it slightly overestimated (mean bias of from −9.87 to −6.03 g/kg DM) the PRCP supply of tropical forage legumes at Kp of 5 and 8%/h.

The underestimation of the PRCP supply of forage grasses for all Kp and forage legumes at slow Kp by the in vitro method can be explained by microbial lysis takes place in a close in vitro system, because rumen microbes lack sufficient substrate for continued fermentation. In the same line, overestimation of the PRCP supply from forage legumes at short incubation times (i.e., fast Kp) might be explained by early microbial lysis in the blank [8,49], which does not occur in the syringes filled with feed substrate. 

In the present study, the adequacy of the in vitro method was considered unacceptable for tropical forages, because it could not reach a CCC of ≥0.80. Such a threshold to decide whether a method allows for predictions with acceptable accuracy and precision will certainly depend on the purpose of its use. Moreover, the estimated CCC of the conjoint sample set was greater than 0.69 for Kp of 5 and 8%/h but not for Kp of 2%/h. These results suggest that the in vitro method can be used as an alternative method to estimate PRCP supply in diets with moderate to fast Kp (e.g., moderate to high feed intake levels) but not with very slow Kp.

### 4.4. Adequacy of the Estimates of Post-Ruminal Crude Protein Supply Using the Chemical Method

In the present study, the CCC of the correlations between the PRCP supply at Kp of 5%/h from tropical forage grasses and forage legumes (n = 38) estimated with the equation of Lebzien et al. [15] and those predicted with the chemical method using the equation of Zhao and Cao [5] suggested a poor level of agreement (CCC from 0.03 to 0.14).

The equation of Zhao and Cao [5] was used in the present study because their equation was developed to estimate the PRCP supply of dried forage grasses, a grass silage, a fresh forage legume, and corn and soybean by-products, whereas the equations of Westreicher-Kristen et al. [7] were specifically developed to predict the PRCP supply of dried distillers’ grains with solubles. Yet, this poor level of agreement was expected, mainly because only a few forage samples (n = 6) and of them only one fresh forage sample (i.e., *Medicago sativa* L.) was included in their sample set that was also used in domestic cattle feeding in the tropics and subtropics. Moreover, their mean MAPE was greater for forage samples (i.e., MAPE of 22%) than for by-product feeds (i.e., MAPE of 9%), which suggests that this equation may perform better for by-products than for forages.

The PRCP supply determined according to Zhao and Cao [5] greatly overestimated the PRCP supply at Kp of 5%/h for both, forage grasses and legumes, and the low CCC was mainly due to a low accuracy (Cb from 0.05 to 0.16) rather than a poor precision (*p* from 0.56 to 0.87). Accordingly, Zhao and Lebzien [6] concluded that the PRCP supply determined after 24 h of in vitro incubation, which was used as reference value by Zhao and Cao [5], overestimates the PRCP supply of forages grasses, although the precision of the predicted PRCP supply of fresh tropical forages calculated with the same sample set used to develop their equations was high. 

The low accuracy of the equation of Zhao and Cao [5] might be due to the fact that tropical forages generally have a slower rate of ruminal CP degradation than temperate ones [10], which may alter the relationships between independent and dependent variables (i.e., coefficient values). Another possible explanation for the discrepancies between PRCP supply predicted either by the equation of Lebzien et al. [15] or of Zhao and Cao [5] could be related to the fact that the latter equation was developed using the PRCP supply determined in vitro as a reference, which itself has its inherent errors (i.e., expected MAPE between measured and in vitro estimated PRCP from 12 to 17% depending on the Kp) as previously discussed in Section 4.3 of the present study. 

In the present study, adequacy of the chemical method was considered unacceptable for tropical forages, because it could not reach a CCC of ≥ 0.80; however, as this low adequacy was mainly due to a low accuracy, specific equations for tropical forages will likely improve the prediction of PRCP supply from tropical forages using the chemical method.

### 4.5. Prediction of Post-Ruminal Crude Protein Supply of Tropical Forages 

An attempt was made to develop one equation per Kp and per forage type (i.e., forage grasses and forage legumes) to predict the PRCP supply from the concentrations of proximate nutrient, fiber, and CP fractions; nevertheless, the predictions were more accurate and precise with one general equation across both forage types than the separate specific equations. The poor prediction with specific equations by forage type could be due to the small sample size for either, the forage grasses (n = 23) or forage legumes (n = 15). Moreover, the chemical composition of the forages varies greatly amongst different species and varieties of tropical forages, even at the same PRCP supply, particularly in the forage legumes [9] as also shown by numerical differences in the present study, which hampers prediction of PRCP supply. 

The independent variables selected in the present study to predict the PRCP supply of tropical forages at Kp of 2, 5 and 8%/h were the concentrations of IP (i.e., sum of true protein fractions B_2_, B_3_, and C) and of ADF. The same independent variables were retained in the equations of Westreicher-Kristen et al. [7] developed to predict the PRCP supply of distillers’ grains. Instead, the equation of Zhao and Cao [5] only included concentrations of all CP fractions.

The concentration of IP explained the greatest proportion of the variance in the PRCP supply of fresh tropical forages as estimated with the equation of Lebzien et al. [15], which is likely related to the significant contribution of the CP fractions B_2_, B_3_, and C to total PRCP supply. The undegraded proportions of the true protein fractions B_2_ and B_3_, with variable rumen degradability, are a considerable part of the RUP [5], and the true protein fraction C is assumed not to be degraded at all within the rumen [12]. Accordingly, the concentrations of the true protein fractions B_3_ and C and their sum are the most important predictors of the RUP concentrations [53,54,55,56] and thus PRCP supply [7] in ruminant forages. The concentration of ADF is also a good predictor of the concentration of RUP [57], with greater ADF concentrations resulting in greater RUP supply from ruminant feedstuffs. Nevertheless, the negative relationship observed between forage ADF concentrations and PRCP supply in the present study is likely related to the fact that greater ADF concentrations strongly reduce DOM, which is in turn highly correlated with microbial CP synthesis [15] as a major constituent of PRCP.

In the present study, an attempt was also made to develop an equation to predict the PRCP supply according to Lebzien et al. [15] from the net NH_3_-N release after 8 and 48 h during in vitro incubation and PRCP supply at Kp of 2, 5, and 8%/h determined by the in vitro method by using linear regression. The NH_3_-N release after 8 h in vitro incubation explained better the variance in PRCP supply as estimated according to Lebzien et al. [1] R^2^ from 0.64 to 0.71) than the NH_3_-N release after 48 h in vitro incubation (R^2^ from 0.44 to 0.52), whereas the effective in vitro PRCP supply (i.e., PRCP supply at Kp of 2, 5, and 8%/h) determined with the in vitro method explained between 41 to 76% of the variance in PRCP supply (data not shown). Although variables obtained by the in vitro method explained a great proportion of the variance in our reference PRCP supply (R^2^ from 0.41 to 0.76), the IP concentration, as determined by the chemical method, was yet a much better predictor of PRCP supply from tropical forages (R^2^ from 0.78 to 0.83).

The RMSE (5.3 to 5.4%) and adjusted R^2^ (0.80 to 0.85) of the equations developed for tropical forages in the present study, as measures of accuracy and precision, respectively, were lower than those of the equation proposed by Zhao and Cao [5] (RMSE of 12.5% and adjusted R^2^ of 0.95), but within the range of those reported by Westreicher-Kristen et al. [7] (RMSE from 2.3 to 10.2% and adjusted R^2^ from 0.75 to 0.95). These results show the significant relationship between CP fractions and PRCP supply irrespective of the forage type. Nevertheless, their validation using an independent larger dataset on the concentrations of different CP and fiber fractions in a range of tropical forage grasses and legumes and their PRCP supply determined in vivo is still needed.

## 5. Conclusions

The in vitro method can be used as an alternative method to estimate PRCP supply in tropical forages at moderate to fast Kp (e.g., moderate to high feed intake levels) but not at very slow Kp. A lower accuracy and precision of the PRCP supply should be expected in tropical forage legumes than forage grasses. 

Moreover, available regression equations developed for temperate ruminant feedstuffs were not accurate and precise enough to predict the PRCP supply of fresh tropical forages from concentrations of chemical CP fractions. Instead, equations developed in the present study appear to allow for an estimation of the PRCP supply of tropical forage grasses and legumes from fiber and CP fractions with a similar chemical composition than the samples included in the present study with reasonable adequacy. Nevertheless, further research is required to validate these equations in diverse species, origins, and phenological stages of forages used in cattle feeding in the tropics and subtropics.

## Figures and Tables

**Table 1:** Descriptive statistics of the concentrations of proximate nutrients, chemical fiber fractions, crude protein fractions, feed fermentation parameters after 24 h in vitro incubation, as well as the post-ruminal crude protein supply at rumen passage rates of 2, 5, and 8%/h of fresh tropical forage grasses and legumes.

	Tropical Forage Grasses (n = 23)					Tropical Forage Legumes (n = 15)				
	Mean	Median	SD	Min	Max	Mean	Median	SD	Min	Max
Proximate nutrient and chemical fiber fractions [g/kg dry matter]										
Crude ash	123	119	29	76	178	74	70	16	45	99
Crude protein	117	119	34	46	201	177	174	25	135	212
Neutral-detergent fiber ^a^	576	573	41	481	654	448	460	69	328	586
Acid-detergent fiber ^b^	308	304	33	220	359	313	320	59	201	414
Lignin_(sa)_ ^c^	33	30	20	6	93	69	69	19	46	125
NDFp ^d^	677	678	40	592	758	477	459	65	382	585
ADFp ^e^	357	363	33	278	421	356	340	62	269	486
Crude protein fractions [g/kg dry matter] ^f^										
A	43.7	41.0	18.8	15.7	93.6	47.9	42.5	14.1	24.4	75.4
B_1_	3.4	3.3	2.3	0.3	9.2	6.4	5.1	5.0	0.2	17.2
B_2_	23.9	23.1	8.0	11.3	40.4	62.3	65.2	18.5	24.2	95.7
B_3_	32.0	34.0	12.0	6.1	51.8	38.6	39.5	17.7	6.2	69.9
C	14.0	12.6	6.4	5.4	27.0	21.2	16.6	9.6	9.6	47.0
In vitro fermentation parameters (24 h) ^g^										
GP [mL/200 mg dry matter]	29	29	3	24	34	34	33	6	25	43
DOM [g/g dry matter]	0.48	0.48	0.03	0.43	0.53	0.55	0.55	0.05	0.49	0.64
ME [MJ/kg dry matter]	6.73	6.73	0.43	5.81	7.62	8.02	7.97	0.89	6.81	10.01
Post-ruminal crude protein [g/kg dry matter] ^h^										
2%/h	105	107	10	82	119	125	122	9	111	150
5%/h	110	113	12	81	127	132	128	11	117	162
8%/h	113	116	13	81	132	137	133	12	121	171

^a^ Neutral-detergent fiber determined using heat-stable amylase and sodium sulfite and expressed inclusive of residual ash. ^b^ Acid-detergent fiber expressed inclusive of residual ash. ^c^ Acid-detergent lignin assayed using sulfuric acid expressed inclusive ash. ^d^ Neutral-detergent fiber assayed using heat-stable amylase and without the use of sodium sulfite using the crude protein fractionation method and expressed inclusive ash. ^e^ Acid-detergent fiber estimated using the crude protein fractionation method and expressed inclusive ash. ^f^ Crude protein fractions described by Sniffen et al. [12] and analyzed following Licitra et al. [13]: A, crude protein soluble in the borate-phosphate buffer and tungstic acid solution; B_1_, true protein soluble in buffer solution and precipitated by the tungstic solution; B_2_, true protein insoluble in buffer solution but soluble in the neutral-detergent solution; B_3_, true protein soluble in acid-detergent solution but insoluble in neutral-detergent solution; and C, true protein insoluble in the acid-detergent solution. ^g^ GP, gas production obtained from in vitro fermentation using the Hohenheim gas test; DOM, digested organic matter estimated using the equation N°43e [14]. The digested organic matter (g/g organic matter) was then multiplied by the organic matter concentration (g/kg dry matter) of the forage sample and divided by 1000 to obtain digested organic matter (g/g dry matter); ME, metabolizable energy estimated with the equation N°12f [14]. ^h^ Post-ruminal supply determined at rumen passage rates of 2, 5, and 8%/h with the equation N°11 of Lebzien et al. [15] using information on in situ rumen-undegraded crude protein at rumen passage rates of 2, 5, and 8%/h, crude protein, and digested organic matter concentration determined from in vitro gas production.

**Table 2:** Predictions of the post-ruminal crude protein (PRCP) supply as estimated with the reference and in vitro methods at rumen passage rates of 2, 5, and 8%/h and as calculated with the chemical method using the equation of Zhao and Cao [5] at rumen passage rate of 5%/h of fresh tropical forage grasses and legumes.

			Error-Index ^c^			Dimensionless ^d^			
Kp ^a^	PRCP Method ^b^	Mean	Mean Bias	RMSE	MAPE	RSR	Concordance Correlation Coefficient		
[%/h]		[g/kg Dry Matter]	[g/kg Dry Matter]	[% Mean Reference PRCP]	[% Mean Reference PRCP]	[From 0 to ∞]	Coefficient [From −1 to 1]	ρ [From −1 to 1]	Cb [From 0 to 1]
Fresh Tropical Forages (n = 38)									
2	Reference PRCP	113							
	In vitro PRCP	108	5.17	17	14	1.26	0.53	0.65	0.82
5	Reference PRCP	119							
	In vitro PRCP	117	2.83	16	13	0.99	0.69	0.84	0.83
	Chemical PRCP	200	−66.91	74	67	4.68	0.14	0.87	0.16
8	Reference PRCP	122							
	In vitro PRCP	123	1.17	15	13	0.86	0.74	0.88	0.84
Fresh Tropical Forage Grasses (n = 23)									
2	Reference PRCP	105							
	In vitro PRCP	100	5.90	16	13	1.60	0.53	0.75	0.71
5	Reference PRCP	110							
	In vitro PRCP	102	8.61	15	13	1.22	0.66	0.89	0.73
	Chemical PRCP	173	−56.04	62	56	5.05	0.13	0.83	0.16
8	Reference PRCP	113							
	In vitro PRCP	105	8.37	13	12	0.98	0.73	0.93	0.78
Fresh Tropical Forage Legumes (n = 15)									
2	Reference PRCP	125							
	In vitro PRCP	120	4.05	19	14	1.93	0.20	0.30	0.65
5	Reference PRCP	132							
	In vitro PRCP	140	−6.03	16	13	1.46	0.29	0.39	0.73
	Chemical PRCP	242	−83.59	85	84	7.62	0.03	0.56	0.05
8	Reference PRCP	137							
	In vitro PRCP	150	−9.87	17	15	1.33	0.30	0.44	0.68

^a^ Passage rates through the rumen. ^b^ PRCP methods: reference PRCP, PRCP supply determined at rumen passage rates of 2, 5, and 8%/h with the equation N°11 of Lebzien et al. [15] using information on in situ rumen-undegraded crude protein at rumen passage rates of 2, 5 and 8%/h, crude protein, and digested organic matter concentration determined from in vitro gas production; in vitro PRCP, PRCP supply estimated with the in vitro method [4]; chemical PRCP, PRCP supply calculated from concentrations of crude protein fractions using the equation of Zhao and Cao [5] for dried forage grasses, a grass silage, a fresh forage legume, and corn and soybean by-products. Results from the chemical method were only compared at a rumen passage rate of 5%/h, because the method was validated against a PRCP measurement after 24 h in vitro incubation, which resembles a PRCP supply at a rumen passage rate of 5%/h. ^c^ Error-index measurements include measures on mean bias, root mean square error (RMSE), and mean absolute percentage error (MAPE). ^d^ Dimensionless; includes measures such as the ratio between root mean square error and standard deviation (RSR), the concordance correlation coefficient (CCC), and its partitioning into correlation coefficient (ρ, i.e., precision) and bias correction factor (Cb; i.e., accuracy).

**Table 3:** Statistical parameters of multivariate regression models developed to estimate post-ruminal crude protein (PRCP) supply at rumen passage rates of 2, 5, and 8%/h of fresh tropical forage grasses and legumes (n = 38).

Dependent Variables ^a^	Intercept and Independent Variables ^b^	Parameters Estimate	SEM	Value	Adjusted R^2 c^	RMSE ^d^	MAPE ^e^
[g/kg Dry Matter]	[g/kg Dry Matter]					[% Mean Reference PRCP]	[% Mean Reference PRCP]
PRCP	Intercept	94.96	8.23	<0.01	0.80	5.29	4.25
Kp 2%/h	B_2_ + B_3_ + C	0.36	0.03	<0.01			
	ADF	−0.05	0.02	0.05			
PRCP	Intercept	97.45	8.66	<0.01	0.82	5.31	4.37
Kp 5%/h	B_2_ + B_3_ + C	0.42	0.03	<0.01			
	ADF	−0.05	0.02	0.03			
PRCP	Intercept	97.52	9.07	<0.01	0.85	5.40	4.41
Kp 8%/h	B_2_ + B_3_ + C	0.47	0.04	<0.01			
	ADF	−0.06	0.02	0.03			

^a^ PRCP supply determined at rumen passage rates of 2, 5, and 8%/h with the equation N°11 of Lebzien et al. [15] using information on in situ rumen-undegraded crude protein at rumen passage rates of 2, 5, and 8%/h, crude protein, and digested organic matter concentration determined from in vitro gas production. ^b^ ADF is the acid-detergent fiber determined in an ANKOM Fiber Analyzer and expressed inclusive ash; B_2_, true protein insoluble in buffer solution but soluble in the neutral-detergent solution; B_3_, true protein soluble in acid-detergent solution but insoluble in neutral-detergent solution; and C, true protein insoluble in the acid-detergent solution. ^c^ Coefficient of determination adjusted by the number of predictors in the model. ^d^ Root mean square error. ^e^ Mean absolute percentage error.

**Figure A1:**
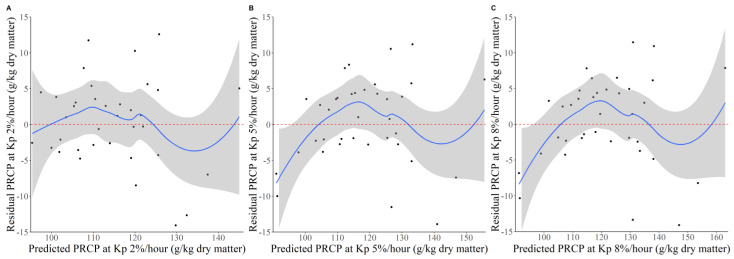
Residuals between post-ruminal crude protein (PRCP) supply estimated with Lebzien et al. [15] equation and PRCP predicted with the developed model versus predicted PRCP supply of tropical forage grasses and legumes at rumen passage rates (Kp) of 2 (**A**), 5 (**B**) and 8 %/h (**C**).

## Appendix A

**Table A1:** Concentrations of crude protein fractions and post-ruminal crude protein supply determined using Lebzien et al. [15] equation of forages commonly used in tropical and subtropical ruminant husbandry systems at rumen passage rates of 2, 5, and 8%/h (all in g/kg dry matter).

Forage Samples	Origin ^a^	Season ^b^	Crude Protein Fractions ^c^					Post-Ruminal Crude Protein ^d^		
			A	B_1_	B_2_	B_3_	C	Kp 2%/h	Kp 5%/h	Kp 8%/h
Fresh tropical forage grasses										
*Andropogon gayanus* Kunth	ES	RS	20.3	4.3	13.4	34.0	8.6	97	101	103
*Andropogon gayanus* Kunth	KY	DS	22.2	1.7	11.3	32.5	17.4	98	102	103
*Brachiaria brizantha* (Hochst. ex A. Rich.) Stapf	PE	RS	47.9	4.1	27.1	18.3	12.6	100	104	105
*Brachiaria brizantha* (Hochst. ex A. Rich.) Stapf *x Brachiaria ruziziensis* R. Germ. and C.M. Evrard	KY	DS	15.7	3.3	15.6	6.1	5.4	82	81	81
*Cenchrus ciliaris* L.	KY	DS	39.8	4.1	35.9	35.4	24.7	119	127	130
*Chloris gayana* Kunth	KY	DS	41.0	3.9	25.4	41.8	20.8	116	123	127
*Cynodon dactylon* (L.) Pers.	KY	DS	54.7	4.9	22.9	45.9	11.1	114	120	123
*Cynodon nlemfuensis* Vanderyst	KY	DS	77.8	0.5	21.3	34.2	15.7	116	120	123
*Digitaria decumbens* Stent	PE	RS	29.2	4.4	21.0	12.5	8.2	93	94	95
*Digitaria eriantha* Steud.	KY	DS	44.1	4.5	13.6	36.1	12.9	104	109	112
*Eragrostis echinochloidea* Stapf	KY	DS	57.6	1.4	19.1	31.7	10.0	105	107	109
*Hyparrhenia rufa* (Nees) Stapf	ET	RS	34.9	0.8	29.2	39.9	11.4	107	113	117
*Hyparrhenia rufa* (Nees) Stapf	KY	DS	19.7	2.0	18.8	28.2	27.0	108	113	116
*Melinis minutiflora* P. Beauv.	KY	DS	29.1	4.7	16.9	39.5	22.3	102	109	113
*Panicum coloratum* L.	ET	RS	69.2	8.9	31.8	33.7	6.6	115	119	121
*Panicum coloratum* L.	KY	DS	56.7	1.2	23.1	44.6	14.4	111	117	121
*Panicum maximum* Jacq.	PE	RS	26.9	3.0	13.3	9.8	7.1	84	84	84
*Pennisetum clandestinum* Hochst. ex Chiov.	ET	RS	93.6	9.2	37.1	51.8	8.8	112	115	118
*Pennisetum clandestinum* Hochst. ex Chiov.	KY	DS	40.2	2.5	40.4	42.1	21.1	114	124	128
*Pennisetum pedicellatum* Trin.	ET	RS	50.4	5.4	27.8	34.0	7.8	103	108	111
*Pennisetum purpureum* Schumach.	ET	RS	51.4	0.3	27.5	32.2	8.1	109	114	117
*Pennisetum purpureum* Schumach.	KY	DS	38.3	1.9	23.7	12.1	15.7	102	104	105
*Tripsacum andersonii* J. R. Gray	KY	DS	43.7	1.8	33.5	39.7	23.8	117	127	132
Fresh tropical forage legumes										
*Arachis glabrata* Benth.	ES	RS	24.4	12.9	48.7	47.8	18.1	130	139	144
*Arachis pintoi* Krapov. and W. C. Greg.	BR	DS	66.2	2.2	53.8	23.0	28.9	123	127	130
*Calopogonium mucunoides* Desv.	BR	DS	57.4	4.3	81.6	33.9	16.6	116	122	126
*Canavalia ensiformis* (L.) DC.	ES	RS	61.0	1.8	65.2	40.4	16.4	121	128	133
*Centrosema sp.* (DC.) Benth.	BR	DS	35.7	8.6	61.4	69.9	27.9	130	139	145
*Crotalaria longirostrata* Hook. and Arn.	ES	RS	42.5	1.9	74.6	6.2	9.6	121	122	123
*Dolichos lablab* L.	ES	RS	38.6	1.7	59.6	38.0	16.2	130	137	142
*Glycine max* (L.) Merr.	ES	RS	75.4	17.2	95.7	12.1	10.8	138	144	149
*Giricidia sepium* (Jacq.) Kunth	ES	RS	47.6	0.2	67.4	65.5	31.1	150	162	171
*Macroptilium atropurpureum* (DC.) Urb.	ES	RS	42.3	11.0	71.1	57.8	15.6	120	127	133
*Mucuna pruriens* (L.) DC.	ES	RS	30.4	5.1	24.2	49.9	47.0	119	127	132
*Pueraria phaseoloides* (Roxb.) Benth.	PE	RS	65.4	6.1	85.2	21.5	15.9	129	134	138
*Stylosanthes guianensis* (Aubl.) Sw.	ES	RS	37.7	12.2	46.1	42.5	19.1	121	129	135
*Stylosanthes guianensis* (Aubl.) Sw.	BR	DS	42.2	9.2	33.1	39.5	30.4	111	117	121
*Vigna sinensis* (L.) Savi ex Hassk.	PE	RS	51.1	2.3	66.8	30.5	14.4	122	126	130

^a^ BR, Brazil; CR, Costa Rica; ES, El Salvador; ET, Ethiopia; KE, Kenya; PE, Peru. ^b^ DS, dry season; RS, rainy season. ^c^ Crude protein fractions described by Sniffen et al. [2] and analyzed following Licitra et al. [3]: A, crude protein soluble in the borate-phosphate buffer and tungstic acid solution; B_1_, true protein soluble in buffer solution and precipitated by the tungstic solution; B_2_, true protein insoluble in buffer solution but soluble in the neutral-detergent solution; B_3_, true protein soluble in acid-detergent solution but insoluble in neutral-detergent solution; C, true protein insoluble in the acid-detergent solution. ^d^ Post-ruminal crude protein supply determined at rumen passage rates of 2, 5, and 8 %/h with the equation N°11 of Lebzien et al. [15] using information on in situ rumen-undegraded crude protein at rumen passage rates of 2, 5, and 8 %/h, crude protein, and digested organic matter concentration determined from in vitro gas production.
