# Correction: Salazar-Cubillas, K.C.; Dickhoefer, U. Evaluating the Protein Value of Fresh Tropical Forage Grasses and Forage Legumes Using In Vitro and Chemical Fractionation Methods. *Animals* 2021, *11*, 2853

**DOI:** 10.3390/ani12020173

**Published:** 2022-01-12

**Authors:** Khaterine C. Salazar-Cubillas, Uta Dickhoefer

**Affiliations:** 1 Institute of Agricultural Sciences in the Tropics (Hans-Ruthenberg-Institute), Animal Nutrition and Rangeland Management in the Tropics and Subtropics, University of Hohenheim, 70599 Stuttgart, Germany; khaterine.salazar-cubillas@uni-hohenheim.de; 2 Institute of Animal Nutrition and Physiology, Christian-Albrechts-Universität zu Kiel, 24118 Kiel, Germany

## Error in Figure/Table

The authors wish to make the following correction to their paper [1]. In the Results section, Table 2 was incorrect due to some errors with the location of numbers. The correct version of the table appears below. The authors apologize for any inconvenience; the change does not affect the scientific results on the animal level.

The authors would like to apologize for any inconvenience to the readers caused by these errors.

## Figures and Tables

**Table 2:** Predictions of the post-ruminal crude protein (PRCP) supply as estimated with the reference and in vitro methods at rumen passage rates of 2, 5, and 8%/h and as calculated with the chemical method using the equation of Zhao and Cao [5] at rumen passage rate of 5%/h of fresh tropical forage grasses and legumes.

			Error-Index ^c^			Dimensionless ^d^			
Kp ^a^	PRCP Method ^b^	Mean	Mean Bias	RMSE	MAPE	RSR	Concordance Correlation Coefficient		
[%/h]		[g/kg Dry Matter]	[g/kg Dry Matter]	[% Mean Reference PRCP]	[% Mean Reference PRCP]	[From 0 to ∞]	Coefficient [From −1 to 1]	ρ [From −1 to 1]	Cb [From 0 to 1]
Fresh Tropical Forages (n = 38)									
2	Reference PRCP	113							
	In vitro PRCP	108	5.17	17	14	1.26	0.53	0.65	0.82
5	Reference PRCP	119							
	In vitro PRCP	117	2.83	16	13	0.99	0.69	0.84	0.83
	Chemical PRCP	200	−66.91	74	67	4.68	0.14	0.87	0.16
8	Reference PRCP	122							
	In vitro PRCP	123	1.17	15	13	0.86	0.74	0.88	0.84
Fresh Tropical Forage Grasses (n = 23)									
2	Reference PRCP	105							
	In vitro PRCP	100	5.90	16	13	1.60	0.53	0.75	0.71
5	Reference PRCP	110							
	In vitro PRCP	102	8.61	15	13	1.22	0.66	0.89	0.73
	Chemical PRCP	173	−56.04	62	56	5.05	0.13	0.83	0.16
8	Reference PRCP	113							
	In vitro PRCP	105	8.37	13	12	0.98	0.73	0.93	0.78
Fresh Tropical Forage Legumes (n = 15)									
2	Reference PRCP	125							
	In vitro PRCP	120	4.05	19	14	1.93	0.20	0.30	0.65
5	Reference PRCP	132							
	In vitro PRCP	140	−6.03	16	13	1.46	0.29	0.39	0.73
	Chemical PRCP	242	−83.59	85	84	7.62	0.03	0.56	0.05
8	Reference PRCP	137							
	In vitro PRCP	150	−9.87	17	15	1.33	0.30	0.44	0.68

^a^ Passage rates through the rumen. ^b^ PRCP methods: reference PRCP, PRCP supply determined at rumen passage rates of 2, 5, and 8%/h with the equation N°11 of Lebzien et al. [15] using information on in situ rumen-undegraded crude protein at rumen passage rates of 2, 5 and 8%/h, crude protein, and digested organic matter concentration determined from in vitro gas production; in vitro PRCP, PRCP supply estimated with the in vitro method [4]; chemical PRCP, PRCP supply calculated from concentrations of crude protein fractions using the equation of Zhao and Cao [5] for dried forage grasses, a grass silage, a fresh forage legume, and corn and soybean by-products. Results from the chemical method were only compared at a rumen passage rate of 5%/h, because the method was validated against a PRCP measurement after 24 h in vitro incubation, which resembles a PRCP supply at a rumen passage rate of 5%/h. ^c^ Error-index measurements include measures on mean bias, root mean square error (RMSE), and mean absolute percentage error (MAPE). ^d^ Dimensionless; includes measures such as the ratio between root mean square error and standard deviation (RSR), the concordance correlation coefficient (CCC), and its partitioning into correlation coefficient (ρ, i.e., precision) and bias correction factor (Cb; i.e., accuracy).
